# *Gardenia jasminoides* Extract Attenuates the UVB-Induced Expressions of Cytokines in Keratinocytes and Indirectly Inhibits Matrix Metalloproteinase-1 Expression in Human Dermal Fibroblasts

**DOI:** 10.1155/2014/429246

**Published:** 2014-03-11

**Authors:** Jiaa Park, Jin Kyung Seok, Hwa-Jin Suh, Yong Chool Boo

**Affiliations:** ^1^Department of Molecular Medicine, Cell and Matrix Research Institute, BK21 Plus KNU Biomedical Convergence Program, Kyungpook National University School of Medicine, Daegu 700-422, Republic of Korea; ^2^Gyeongbuk Natural Color Industry Institute, Gyeongbuk, Republic of Korea; ^3^Ruby Crown Co., Ltd., Daegu, Republic of Korea

## Abstract

Ultraviolet radiation (UV) is a major cause of photoaging, which also involves inflammatory cytokines and matrix metalloproteinases (MMP). The present study was undertaken to examine the UVB-protecting effects of yellow-colored plant extracts in cell-based assays. HaCaT keratinocytes were exposed to UVB in the absence or presence of plant extracts, and resulting changes in cell viability and inflammatory cytokine expression were measured. Of the plant extracts tested, *Gardenia jasminoides* extract showed the lowest cytotoxicity and dose-dependently enhanced the viabilities of UVB-exposed cells. *Gardenia jasminoides* extract also attenuated the mRNA expressions of interleukin-1**β** (IL-1**β**) and tumor necrosis factor-**α** (TNF-**α**) in HaCaT cells stimulated by UVB. Conditioned medium from UVB-exposed HaCaT cells was observed to stimulate MMP-1 protein expression in human dermal fibroblasts, and this effect was much smaller for the conditioned medium of HaCaT cells exposed to UVB in the presence of *Gardenia jasminoides* extract. *Gardenia jasminoides* extract also exhibited antioxidative and antiapoptotic effects in HaCaT cells exposed to UVB. These results indicated that UVB-induced injury and inflammatory responses of skin cells can be attenuated by yellow-colored plant extracts, such as *Gardenia jasminoides* extract.

## 1. Introduction 

Cosmetics have become essential products as people pursue esthetic desires in modern society, and their role has been extended to the retardation of skin aging caused by physiological and environmental factors. Ultraviolet radiation (UV) is a major cause of skin aging, which is characterized by wrinkles, laxity, blister formation, roughness, and loss of skin tone [1–3]. Therefore, there is considerable interest in the development of safer, more effective ingredients that mitigate the harmful effects of UV.

Photoaging involves changes in dermal extracellular matrix composition and collagen loss. Matrix metalloproteinases (MMPs), a group of zinc endopeptidases, play a key role in the turnover of extracellular matrix macromolecules, including type I collagen [4, 5]. Furthermore, the expressions of MMPs, such as MMP-1, -2, -3, and -9, are known to be upregulated in UV-exposed human dermal fibroblasts [6, 7]. These MMPs can regulate the remodeling of connective tissues associated with the formation of wrinkles and other phenotypes of photoaging. Thus, the pharmacological targeting of MMPs is considered a promising strategy to reduce the photoaging process [8].

The UVB-induced upregulation of MMPs is mainly mediated by signal transduction pathways involving the UV-induced activation of cytokine receptors and the subsequent activations of mitogen-activated protein kinases, such as extracellular signal-regulated kinase, c-Jun-N-terminal kinase, and p38 kinase, which in turn leads to the activations of transcription factors, such as activator protein-1 (AP-1) complexes [9–11]. In fact, the promoters of MMP-1 and MMP-3 genes carry AP-1 sites that are transactivated by the binding of active AP-1 complexes, which results in the initiation of MMP gene transcription [10, 12]. Although the initial events underlying this signal cascade are not fully understood, available evidence supports the notion that UV-damaged DNA acts as a primary signal [13].

The communication between epidermal keratinocytes and dermal fibroblasts is important in terms of skin response to various external stimuli, including UV. MMP-1 expression in dermal fibroblasts can be stimulated by direct exposure of the fibroblasts to UV. In addition, MMP-1 expression in dermal fibroblasts can be stimulated indirectly by the cytokines or other factors secreted by epidermal keratinocytes exposed to UV. Previous studies have shown that the conditioned medium from UV irradiated keratinocytes effectively increases MMP-1 expression in fibroblasts via paracrine effects [14, 15]. Thus, MMP-1 expression in dermal fibroblasts may be regulated indirectly by inhibiting the activation of epidermal keratinocytes in response to UV. In this regard, plant extracts with UV-shielding, antioxidative, and anti-inflammatory properties are an attractive prospect [16, 17].

The purpose of the present study was to examine the protective effects of plant extracts derived from* Gardenia jasminoides*,* Phellodendron amurense*, and* Rheum rhabarbarum* against skin cell responses to UV. These yellow plant extracts were chosen because of their ability to absorb UV effectively.

## 2. Materials and Methods

### 2.1. Plant Materials

Extracts of* Gardenia jasminoides Ellis* (seed),* Phellodendron amurense Rupr*. (root), and* Rheum rhabarbarum L*. (root) were prepared at Gyeongbuk Natural Color Industry Institute (http://www.gnc.re.kr/). Voucher specimens of the plants and other information regarding the extracts are available at this institute. Plant materials were extracted with hot water 80°C for 2 h and extracts were evaporated to dryness under reduced pressure.

### 2.2. UV Spectrophotometry

Test materials were dissolved in phosphate buffered saline (PBS) to final concentrations of 10, 30, and 100 *μ*g mL^−1^. Absorption spectra were recorded in the 200~600 nm range using a Shimadzu UV-1650PC spectrophotometer (Shimadzu Corporation, Kyoto, Japan).

### 2.3. HPLC Analysis

HPLC analysis of extracts was done using a Gilson HPLC system (Gilson, Inc., Middleton, WI, USA) equipped with a 321 pump and UV/VIS 151 detector. Aqueous solution of test materials (1.0 mg mL^−1^) was injected at 10 *μ*L. Separation was done on a 5 *μ*m Hector-M C18 column (4.6 mm × 250 mm) (RS Tech Co., Daejeon, Korea). The mobile phase consisted of 0.5% formic acid (A) and acetonitrile (B). The gradient was programmed as follows: 0–20 min, a linear gradient from 20 to 80% B; 20–35 min, 80% B. The flow rate was 0.5 mL min^−1^. The UV detector was set at 280 nm and 440 nm. Purified crocin was purchased from Sigma-Aldrich (St. Louis, MO, USA) and its purity was determined spectrophotometrically using the extinction coefficient *ε*_443_ = 89,000 M^−1^ cm^−1^ [18].

### 2.4. HaCaT Cell Culture

HaCaT cells (a human immortalized keratinocyte cell-line) were grown in DMEM/F-12 medium (GIBCO-BRL, Grand Island, NY, USA) supplemented with a 10% fetal bovine serum, 100 U mL^−1^ penicillin, 0.1 mg mL^−1^ streptomycin, 0.25 *μ*g mL^−1^ amphotericin B, and 10 *μ*g mL^−1^ hydrocortisone. Cells were cultured at 37°C in a humidified atmosphere containing 5% CO_2_ and 95% air.

### 2.5. UVB-Exposure of HaCaT Cells

HaCaT cells were seeded on a six-well plate at a density of 2 × 10^5^ cells per well and grown in a culture medium for 48 h to reach 80% confluency. The cells were then washed twice with PBS and exposed to UVB in PBS containing the test material. UVB treatment was conducted under a cell culture hood using a UVB lamp (Model UVB-18, ULTRA∗LUM. Inc., Claremont, CA, USA) that emitted radiation in the wavelength range of 280 to 340 nm with maximum intensity at 300 nm. The intensity of radiation was determined using a UV light meter (Model UV 340A, Lutron Electronic Enterprise Co., Taipei, Taiwan), and the UV was administered to cells in culture plates at an intensity of 80 *μ*W cm^−2^. However, durations of treatment varied to provide specific doses of UVB (5, 10, or 15 mJ cm^−2^). Following irradiation, PBS was replaced by growth medium and cells were incubated for 1 day. Cells were subjected to various tests as detailed below. Culture conditioned medium was harvested and used to treat human dermal fibroblasts.

### 2.6. Cell Viability Test

Cell viability was assayed using 3-[4,5-dimethylthiazol-2-yl]-2,5-diphenyltetrazolium bromide (MTT). This assay method is based on the ability of mitochondrial dehydrogenase in viable cells to reduce pale yellow MTT to dark blue formazan crystals that accumulate within cells. Briefly, cells were washed with PBS and incubated in 1 mL of culture media supplemented with 1 mg mL^−1^ MTT for 3 h. The medium was then discarded and cells were treated with isopropanol to solubilize the formazan. Solutions were then transferred to microplates and formazan was quantified by measuring absorbance at 595 nm.

### 2.7. Quantitative Polymerase Chain Reaction (qRT-PCR) Analysis

Total cellular RNA was extracted using the RNeasy kit (Qiagen, Valencia, CA, USA). To prepare cDNA, one *μ*g of cellular mRNA was reverse transcribed using the High Capacity cDNA Archive Kit (Applied Biosystems, Foster City, CA, USA). This kit utilizes random hexamers primers and MultiScribe Reverse Transcriptase. PCR was conducted using the StepOnePlus Real-Time PCR System (Applied Biosystems) in reaction mixtures (20 *μ*L) containing SYBR Green PCR Master Mix (Applied Biosystems), 60 ng of cDNA, and 2 picomole of gene-specific primer sets (Macrogen, Seoul). The primers used for PCR analysis were as follows: interleukin-1*β* (IL-1*β*) (GeneBank accession number: NM_000576.2) 5′-CCT GTC CTG CGT GTT GAA AGA-3′ (forward) and 5′-GGG AAC TGG GCA GAC TCA AA-3′ (reverse), tumor necrosis factor-*α* (TNF-*α*) (NM_000594.3) 5′-TGC TCC TCA CCC ACA CCA T-3′ (forward) and 5′-GAG ATA GTC GGG CCG ATT GA-3′ (reverse), and GAPDH (NM_002046.3) 5′-ATG GGG AAG GTG AAG GTC G-3′ (forward) and 5′-GGG GTC ATT GAT GGC AAC AA-3′ (reverse). Reactions were performed using the following conditions: 50°C for 2 min, 95°C for 10 min, and 40 amplification cycles (95°C for 15 s and 60°C for 1 min), followed by a dissociation step. Melting curve analysis showed single peaks, which supported the homogeneity of amplicons. The mRNA expression levels of IL-1*β* and TNF-*α* relative to that of internal control, glyceraldehyde 3-phosphate dehydrogenase (GAPDH), were calculated using the comparative threshold cycle (CT) method.

### 2.8. Culture of Human Dermal Fibroblasts

Human dermal fibroblasts isolated from adult skin were obtained from Cascade Biologics (Portland, OR, USA). The cells were cultured at 37°C in a humidified atmosphere containing 5% CO_2_ and 95% air in growth medium (Iscove's Modified Dulbecco's medium (GIBCO-BRL, Grand Island, NY, USA) containing 10% fetal bovine serum, 100 U mL^−1^ penicillin, 0.1 mg mL^−1^ streptomycin, and 0.25 *μ*g mL^−1^ amphotericin B). In experiments on the paracrine effects of factors secreted by keratinocytes, the medium was completely replaced by the conditioned medium of UVB-irradiated HaCaT cells, and fibroblasts were cultivated for 24 h before analysis.

### 2.9. Western Blotting

Whole cell lysates were prepared using a lysis buffer (10 mM Tris-Cl, pH 7.4, 120 mM NaCl, 25 mM KCl, 2 mM EGTA, 1 mM EDTA, 0.5% Triton X-100, and protease inhibitor cocktail). Aliquots of lysates were subjected to sodium dodecyl sulfate polyacrylamide gel electrophoresis (SDS-PAGE) under protein denaturing conditions. Proteins separated were transferred to polyvinylidene fluoride membranes, which were incubated with an appropriate primary antibody overnight at 4°C and then with a secondary antibody conjugated to horseradish peroxidase for 1 h at room temperature. Immunoreactive bands were detected using a picoEPD Western Reagent kit (ELPIS-Biotech, Daejeon, Korea) and subjected to densitometric analysis. The primary antibody for MMP-1 was purchased from Calbiochem (San Diego, CA, USA). Rabbit polyclonal caspase-3 antibody and rabbit polyclonal caspase-9 were purchased from Cell Signaling (Danvers, MA, USA). The mouse monoclonal *β*-actin antibody was from Sigma-Aldrich (St. Louis, MO, USA).

### 2.10. Analysis of Lipid Peroxidation

As a marker of lipid peroxidation, 2-thiobarbituric acid-reactive substances (TBARS) were quantified [19]. Briefly, cells were treated in a lysis buffer (20 mM Tris-Cl, 2.5 mM EDTA, 1.0% SDS, pH 7.5). Cell lysates (200 mg protein in 100 *μ*L) were mixed with 900 *μ*L of 1.0% phosphoric acid and 1.0 mL of 0.9% 2-thiobarbituric acid (Sigma-Aldrich) and then heated on a boiling water bath for 45 min. Standard solutions of 1,1,3,3-tetramethoxypropane (Sigma-Aldrich), a precursor of malondialdehyde, were treated in the same way as cell lysates. After cooling, 1.5 mL of 1-butanol was added and the mixture was centrifuged at 13,000 rpm for 15 min to separate into two layers. The fluorescence intensity of the 1-butanol layer was measured at an emission wavelength of 590 nm (excitation at 540 nm) using the Gemini EM fluorescence microplate reader (Molecular Devices, Sunnyvale, CA, USA).

### 2.11. Statistical Analysis

Data are presented as the means ± SEs of three or more independent experiments. The significance of differences between groups was determined using the Student's *t*-test. Duncan's multiple-range test was applied when differences were significant (*P* < 0.05) by the Student's *t*-test. Statistical significance was accepted for *P* values < 0.05.

## 3. Results

**Figure 1:**
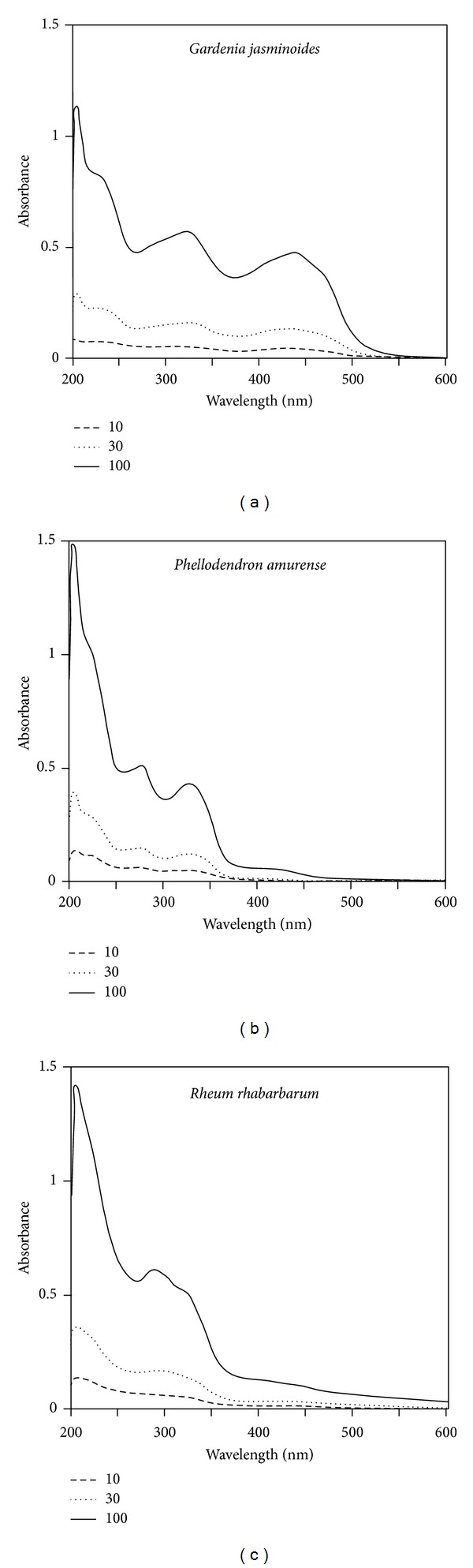
Absorption spectra of plant extracts. Extracts from* Gardenia jasminoides* (a),* Phellodendron amurense* (b), and* Rheum rhabarbarum* (c) were dissolved in PBS at concentrations of 10, 30, or 100 *μ*g mL^−1^.

**Figure 2:**
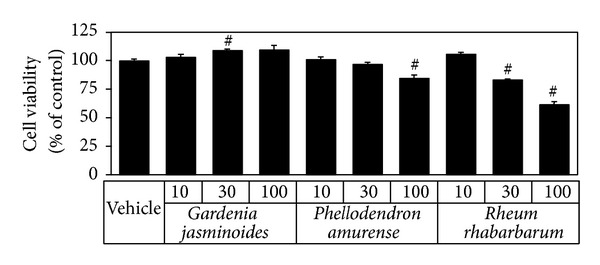
Effects of plant extracts derived from* Gardenia jasminoides, Phellodendron amurense*, and* Rheum rhabarbarum* on the viability of HaCaT keratinocytes. Cells were treated with plant extracts at the indicated concentration for 24 h and cell viabilities were determined using an MTT assay. Cell viabilities are presented as percentages of the vehicle control (means ± SEs; *n* = 3). ^#^ *P* < 0.05* versus* the vehicle control.

The absorption spectra of the extracts of* Gardenia jasminoides, Phellodendron amurense*, and* Rheum rhabarbarum* were measured at 10~100 *μ*g mL^−1^. As shown in Figure 1, these extracts showed similar absorptivities in the UVB region (280~320 nm), and* Gardenia jasminoides* extract showed the highest absorption in the UVA (320~400 nm) and blue light region. The cytotoxicities of extracts were compared by treating HaCaT human keratinocytes at 10~100 *μ*g mL^−1^ for 24 h. As shown in Figure 2,* Gardenia jasminoides* extract appeared to be nontoxic up to 100 *μ*g mL^−1^, whereas* Phellodendron amurense* extract was mildly cytotoxic at this concentration and* Rheum rhabarbarum* was cytotoxic at concentrations above 30 *μ*g mL^−1^.

**Figure 3:**
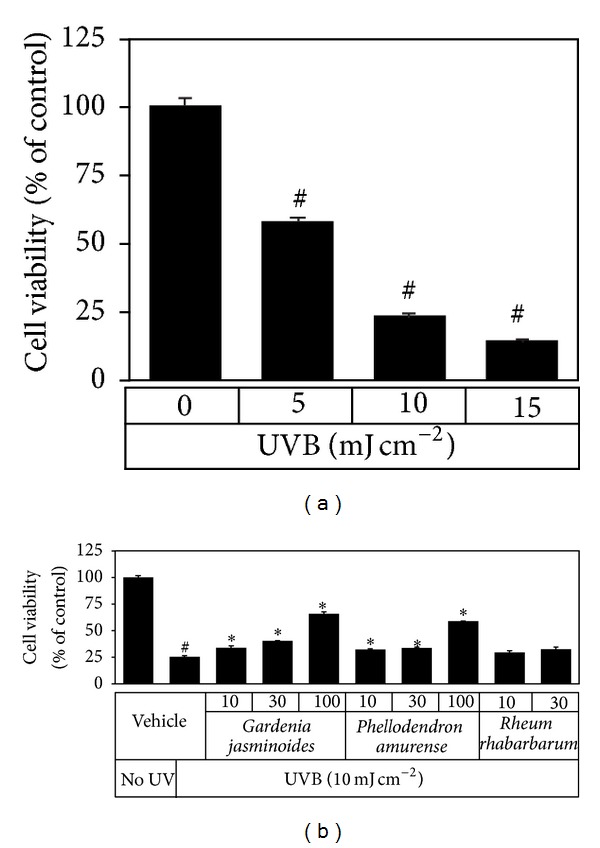
Effects of plant extracts on UVB cytotoxicity in HaCaT cells. In (a), HaCaT cells were irradiated with 5~15 mJ cm^−2^ UVB. In (b), cells were irradiated with 10 mJ cm^−2^ UVB in PBS in the absence or presence of plant extracts (10, 30, or 100 *μ*g mL^−1^). Irradiated cells were subsequently incubated for 24 h and cell viabilities were determined using an MTT assay. Cell viabilities are presented as percentages of the vehicle control (means ± SEs, *n* = 3). ^#^ *P* < 0.05* versus* the unirradiated control. *P < 0.05* versus* the vehicle control irradiated with UVB.

The protective effects of the plant extracts against UVB-induced cell death were then examined. First, HaCaT cells were exposed to 5~15 mJ cm^−2^ and incubated for 24 h, and changes in cell viability were determined. As shown in Figure 3(a), HaCaT cell viability was reduced by UV exposure in a dose-dependent manner. Cells were then exposed to UVB at 10 mJ cm^−2^ in the presence of different concentrations of plant extracts, and cell viabilities were assessed 24 h later. As shown in Figure 3(b),* Gardenia jasminoides* extract dose-dependently increased the viabilities of UVB-exposed cells.* Phellodendron amurense* extract was also found to have a smaller cytoprotective effect, but* Rheum rhabarbarum* extract had no observable effects.

**Figure 4:**
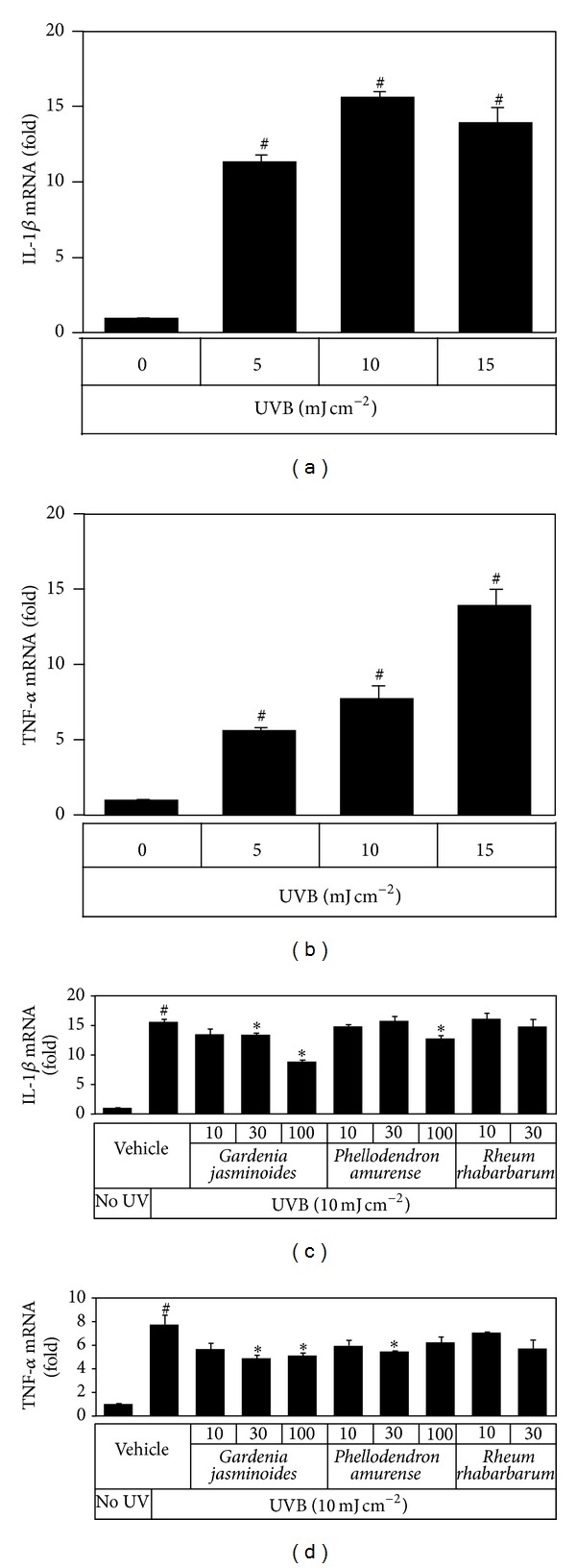
Effects of plant extracts on mRNA expressions of inflammatory cytokines induced by UVB in HaCaT cells. In (a) and (b), HaCaT cells were irradiated with 5~15 mJ cm^−2^ UVB. In (c) and (d), cells were irradiated with 10 mJ cm^−2^ UVB in the absence or presence of plant extracts (10, 30, or 100 *μ*g mL^−1^) and then incubated for 24 h before mRNA analysis by qRT-PCR. GAPDH was used as the reference. Data are expressed as fold changes (means ± SEs; *n* = 3). ^#^ *P* < 0.05* versus* the unirradiated control. *P < 0.05* versus* the vehicle control irradiated with UVB.

The effects of plant extracts on inflammatory cytokine expression were examined in HaCaT cells exposed to UVB at 5~15 mJ cm^−2^ and incubated for 24 h. Total cellular mRNA was extracted and the mRNA levels of cytokines were determined by quantitative Real-Time PCR. As shown in Figure 4(a), UVB increased the mRNA expressions of IL-1*β* and TNF-*α* markedly. Cells were then exposed to UVB at 10 mJ cm^−2^ in the presence of plant extracts at different concentrations. It was found that* Gardenia jasminoides* extract at 30~100 *μ*g mL^−1^ attenuated inflammatory cytokine expression in HaCaT cells exposed to UVB, and* Phellodendron amurense* extract showed a smaller effect.

**Figure 5:**
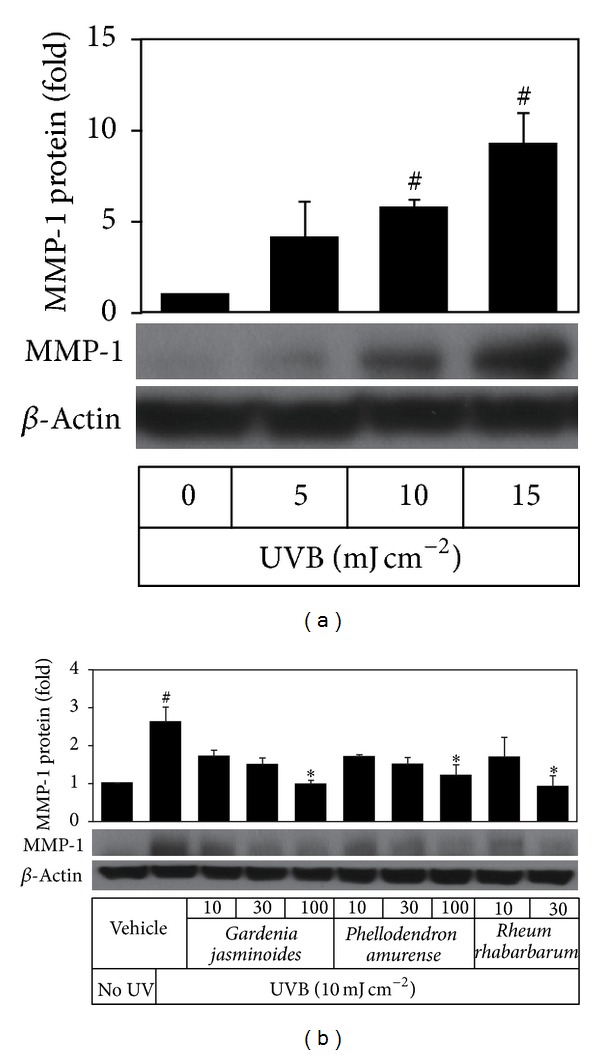
Expression levels of MMP-1 protein in human dermal fibroblasts stimulated by the conditioned media of HaCaT cells irradiated with UVB in the absence or presence of plant extracts. In (a), HaCaT cells were irradiated with UVB at 5~15 mJ cm^−2^ and then incubated for 24 h. The conditioned medium from the UVB-irradiated HaCaT cells was used to treat human dermal fibroblasts for 24 h. In (b), HaCaT cells were irradiated with UVB at 10 mJ cm^−2^ in PBS containing plant extracts at various concentrations  *μ*g mL^−1^ and then incubated for 24 h. Conditioned media from these UVB-irradiated HaCaT cells were used to treat human dermal fibroblasts for 24 h. MMP-1 protein expressions in fibroblasts were analyzed by Western blot using *β*-actin as a loading control. Typical blot images are shown. Data are expressed as fold changes (means ± SEs; *n* = 3). ^#^ *P* < 0.05* versus* the unirradiated control. *P < 0.05* versus* the vehicle control irradiated with UVB.

Because UVB increased inflammatory cytokine expression in HaCaT cells, we examined whether the conditioned medium of UVB-exposed HaCaT keratinocytes, which contained inflammatory cytokines, stimulated MMP-1 expression in fibroblasts* via* paracrine effects. When conditioned medium from HaCaT cells exposed to different doses of UVB was added to human dermal fibroblasts cultures and cultured for 24 h, MMP-1 expressions in fibroblasts were found to increase in a UVB dose-dependent manner (Figure 5(a)). We then examined the effects of plant extracts on the paracrine effects of cytokines secreted by UVB-exposed HaCaT keratinocytes on MMP-1 expression in fibroblasts. As shown in Figure 5(b), the presence of plant extracts such as* Gardenia jasminoides* extract during UVB exposure of HaCaT cells rendered the conditioned medium less effective in increasing MMP-1 expression in fibroblasts.

**Figure 6:**
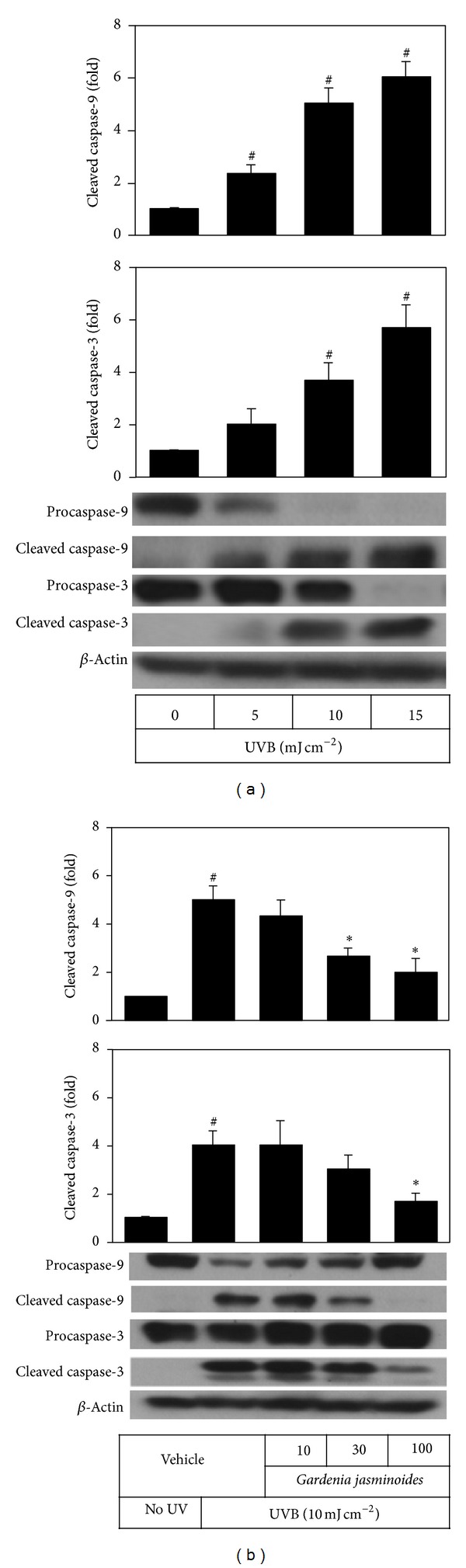
Effects of* Gardenia jasminoides* extract on caspase-9 and caspase-3 in HaCaT cells exposed to UVB radiation. In (a), HaCaT cells were irradiated with 5~15 mJ cm^−2^ UVB. In (b), cells were irradiated with 10 mJ cm^−2^ UVB in the absence or presence of* Gardenia jasminoides* extract (10, 30, or 100 *μ*g mL^−1^) and then incubated for 24 h. The cleavage of procaspase-9 and procaspase-3 to their cleaved active forms was analyzed by Western blot. Data are expressed as fold changes (means ± SEs; *n* = 3). ^#^ *P* < 0.05* versus* the unirradiated control. *P < 0.05* versus* the vehicle control irradiated with UVB.

Apoptotic cell death is also known as one of the major mechanisms of photoaging of the skin [20]. Because caspase-9 and caspase-3 are known to be critical players in UV-induced apoptosis in keratinocytes [21], we examined the effect of* Gardenia jasminoides* extract on the activation of these apoptosis mediator enzymes in HaCaT cells exposed to UVB. As expected, UVB irradiation increased cleavage of procaspase-9 and procaspase-3 to their cleaved active forms in a UVB dose-dependent manner (Figure 6(a)). It was also observed that the UVB-induced increases of cleaved caspase-9 and cleaved caspase-3 were markedly prevented when* Gardenia jasminoides* extract was included in the medium (Figure 6(b)).

**Figure 7:**
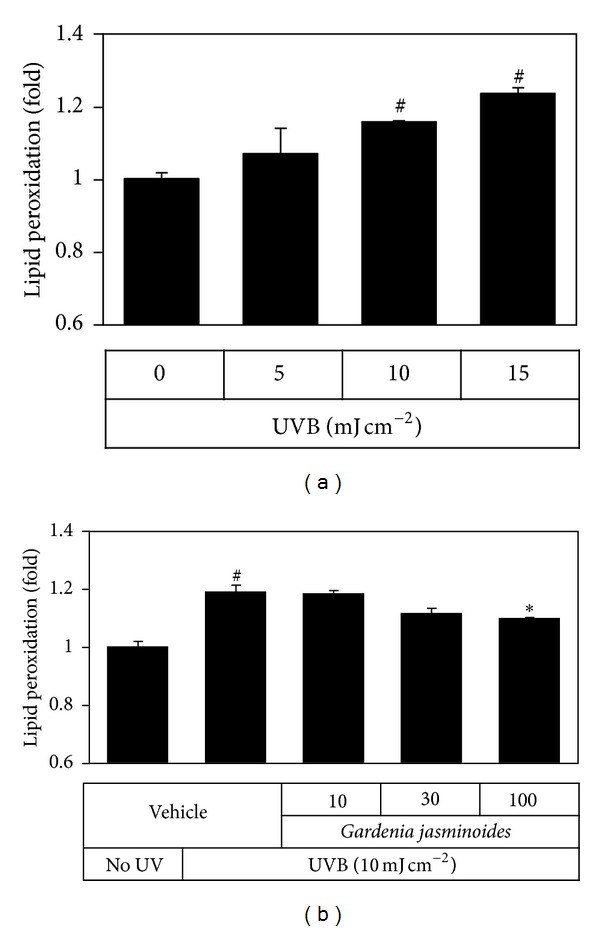
Effects of* Gardenia jasminoides* extract on UVB-induced lipid peroxidation in HaCaT cells. In (a), HaCaT cells were irradiated with 5~15 mJ cm^−2^ UVB. In (b), cells were irradiated with 10 mJ cm^−2^ UVB in the absence or presence of* Gardenia jasminoides* extract (10, 30, or 100 *μ*g mL^−1^) and then incubated for 24 h. Lipid peroxidation was determined by quantifying TBARS. Data are expressed as fold changes (means ± SEs; *n* = 3). Control value was 0.33 nmol TBARS per mg protein. ^#^ *P* < 0.05* versus* the unirradiated control. *P < 0.05* versus* the vehicle control irradiated with UVB.

UV can also induce oxidative stress in cells by stimulating overproduction of reactive oxygen species (ROS) and depleting antioxidants [22, 23]. In the current study, we determined the levels of TBARS to monitor lipid peroxidation [19]. The results showed that lipid peroxidation increased in HaCaT cells exposed to UVB (Figure 7(a)) and this change was attenuated by* Gardenia jasminoides* extract in a dose-dependent manner (Figure 7(b)).

**Figure 8:**
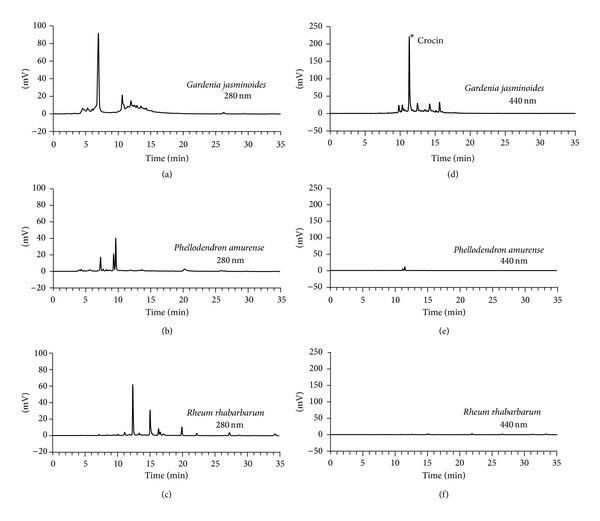
Fingerprint HPLC of plant extracts from* Gardenia jasminoides, Phellodendron amurense*, and* Rheum rhabarbarum*. Typical HPLC chromatograms at 280 nm (a, b, c) and 440 nm (d, e, f) are shown. The peak of crocin was assigned by cochromatography with commercially sourced crocin.

Typical HPLC patterns of the extracts of* Gardenia jasminoides, Phellodendron amurense,* and* Rheum rhabarbarum* are shown in Figure 8. Commercially sourced crocin was used as a reference compound because it is known as a major pigment in* Gardenia jasminoides* [24]. The content of crocin in* Gardenia jasminoides* extract was estimated to be 1.7%. It was not detected in the extract of* Phellodendron amurense* or* Rheum rhabarbarum*.

## 4. Discussion

UV is a major cause of skin photoaging and photocarcinogenesis [25]. UV consists of UVA (320~400 nm), UVB (280~320 nm), and UVC (200~280 nm), and overexposure to UV, particularly the UVB component, causes erythema, edema, hyperplasia, hyperpigmentation, photoaging, immunosuppression, and skin cancer [2, 26]. Overexposure to UV also causes oxidative stress as evidenced by increased lipid peroxidation and by the depletion of cutaneous antioxidants [27, 28]. Previous studies have demonstrated the preventive effects of exogenous antioxidants on photocarcinogenesis [29]. Plant extracts are attractive cosmeceuticals because some are rich in secondary metabolites that provide UVB-screening, antioxidative, and anti-inflammatory activities. The results of the current study indicate that yellow-colored plant extracts like* Gardenia jasminoides* extract have potential use as cosmeceuticals to attenuate the deleterious effects of UV exposure.

Compared to the extracts of* Phellodendron amurense* and* Rheum rhabarbarum*,* Gardenia jasminoides* extract was found to absorb a wider range of UV (Figure 1) and to have minimal effects on the viability of HaCaT cells cultured* in vitro* (Figure 2). In addition,* Gardenia jasminoides* extract attenuated the viability loss of HaCaT cells caused by UVB exposure (Figure 3). Under our experimental conditions, cells were treated with the plant extract in PBS while being exposed to UVB to minimize the UV screening effects of other factors. Thus, we attributed the cytoprotective effect of* Gardenia jasminoides* extract to its UV shielding effects.

Keratinocytes constitute 95% of the mass of human epidermis cells and play critical roles in skin physiology due to their autocrine and paracrine effects [30]. The constitutive productions of cytokines and other soluble factors are low in human keratinocytes, but various stimuli, including endotoxins and UV, can trigger the expression of proinflammatory cytokines [31]. In the present study, UVB exposure increased the expressions of IL-1*β* and TNF-*α* markedly in HaCaT cells, as was expected (Figure 4). In addition, these changes were significantly attenuated by the presence of plant extracts during UV exposure (Figure 4). These results indicate that plant extracts, such as* Gardenia jasminoides* extract, can attenuate cytokine expression in keratinocytes.

MMP-1 expression in human dermal fibroblasts increased when these cells were treated with the conditioned medium from UVB-irradiated HaCaT cells (Figure 5(a)). These results suggest that certain cytokines or cell components from UVB-irradiated keratinocytes can regulate MMP-1 expression in fibroblasts through paracrine effects. Cytokines such as IL-1*β* and TNF-*α* are known to stimulate MMP-1 expression in fibroblasts [32], other factors such as stratifin also could play a role [15, 33]. Nonetheless,* Gardenia jasminoides* extract attenuated these paracrine effects in a dose-dependent manner (Figure 5(b)), which was in line with its effects on cytokine expressions in HaCaT cells (Figure 4).

UV can induce apoptosis of keratinocytes* via* intrinsic pathways involving direct DNA damage, extrinsic pathways involving activated cell membrane death receptors, and generation of ROS [20, 21]. Occurrence of apoptosis can be monitored by using various markers including DNA laddering, changes of proapoptotic (Bax, Bak, and Bid) and antiapoptotic (Bcl-2 and Bcl-x) members of the Bcl-2 protein family, activation of caspases, and so on. In the present study, we observed UVB dose-dependent increases in cleaved active forms of caspase-9 and caspase-3 and attenuation of these changes by* Gardenia jasminoides* extract (Figure 6). In addition,* Gardenia jasminoides* extract was observed to attenuate UVB-induced oxidative stress, as monitored by lipid peroxidation (Figure 7). Therefore,* Gardenia jasminoides* extract was considered to be useful in protecting skin cells from UV-induced oxidative stress, inflammation, and apoptotic death.

*Gardenia jasminoides* belongs to the botanical family* Rubiaceae *and is found in South Asia.* Gardenia jasminoides* fruits have been traditionally used in oriental medicine to treat jaundice, fever, hypertension, and skin ulcers. Its yellow pigment has been used as a natural food colorant and dye. The major constituent of this yellow pigment is crocin, a water soluble carotenoid derivative [24], and the pharmacological effects of crocin, and of its aglycone crocetin, have been widely investigated [34, 35]. The protective effects of* Gardenia jasminoides* extract against UVB-induced injury and inflammatory responses of skin cells might be attributed to the UV shielding effect of crocin (Figures 1 and 8).

## 5. Conclusion

In conclusion, this study suggests that UVB-induced oxidative stress, apoptotic cell death, and inflammatory responses of skin cells can be attenuated by yellow-colored plant extracts, such as* Gardenia jasminoides* extract, that absorb UV effectively, and these extracts are useful cosmetic ingredients.
